# Supplementary material for: Insight into Body Condition Variability in Small Mammals
Source: Animals (Basel). 2024 Jun 5;14(11):1686. doi: 10.3390/ani14111686 (PMC11171198; doi:10.3390/ani14111686)
Supplement: Supplementary file 1 [file animals-14-01686-s001.zip › animals-3025106-supplementary.pdf]

Article

Insight into Body Condition Variability in Small Mammals

Linas Balčiauskas * and Laima Balčiauskienė

Supplemental materials

Table S1. Body condition index statistics in various small mammal species, regardless of age and gender. Superscript letters denote post-hoc tested differences, significant at $p < 0.05$.

Species	N	Mean±SE	Min–max
S. araneus	2303	2.90±0.01 c	1.15–6.25
S. minutus	724	2.88±0.03 c	1.04–5.83
N. fodiens	99	2.71±0.04 d	1.87–4.54
N. milleri	3	2.81±0.34 d	2.13–3.14
M. avellanarius	1	4.47 a	
S. betulina	17	3.34±0.14 b	2.39–4.31
M. musculus	424	3.56±0.03 a	1.70–6.58
A. agrarius	3482	3.20±0.01 b	1.77–6.14
A. flavicollis	5403	3.22±0.01 b	1.50–6.89
A. sylvaticus	4	3.20±0.28 b	2.61–3.95
A. uralensis	68	2.71±0.05 d	1.79–4.08
M. minutus	337	3.73±0.04 a	2.03–6.46
C. glareolus	9866	2.92±0.00 c	1.27–6.51
A. amphibius	10	3.61±0.17 a	3.17–5.05
A. oeconomus	1286	2.89±0.02 c	1.51–6.38
M. arvalis	2429	2.91±0.01 c	1.49–6.23
M. agrestis	652	2.90±0.02 c	1.38–6.10
M. rossiaemeridionalis	30	2.40±0.05 d	1.99–3.30

Table S2. Correlations between body mass (Q) and body length (L) in different age groups of small mammal species, regardless of their gender. Juv – juveniles, sub – subadult, ad – adult animals; *** – correlation significant at $p < 0.001$, ** – $p < 0.01$.

Species	Juv	Sub	Ad
S. araneus	0.37***	0.03^{NS}	0.59***
S. minutus	–0.21^{NS}	0.13^{NS}	0.45***
N. fodiens	0.83***	0.49**	0.13^{NS}
S. betulina	0.93^{NS}	0.89^{NS}	0.84**
M. musculus	0.84***	0.70***	0.83***
A. agrarius	0.70***	0.49***	0.76***
A. flavicollis	0.87***	0.74***	0.78***
A. uralensis	0.66***	0.68***	0.92***
M. minutus	0.48***	0.49***	0.49***
C. glareolus	0.70***	0.39***	0.66***
A. amphibius	0.55^{NS}	0.99**	
A. oeconomus	0.85***	0.69***	0.81***
M. arvalis	0.71***	0.64***	0.74***
M. agrestis	0.70***	0.59***	0.72***
M. rossiaemeridionalis	0.84^{NS}	0.95***	0.93***

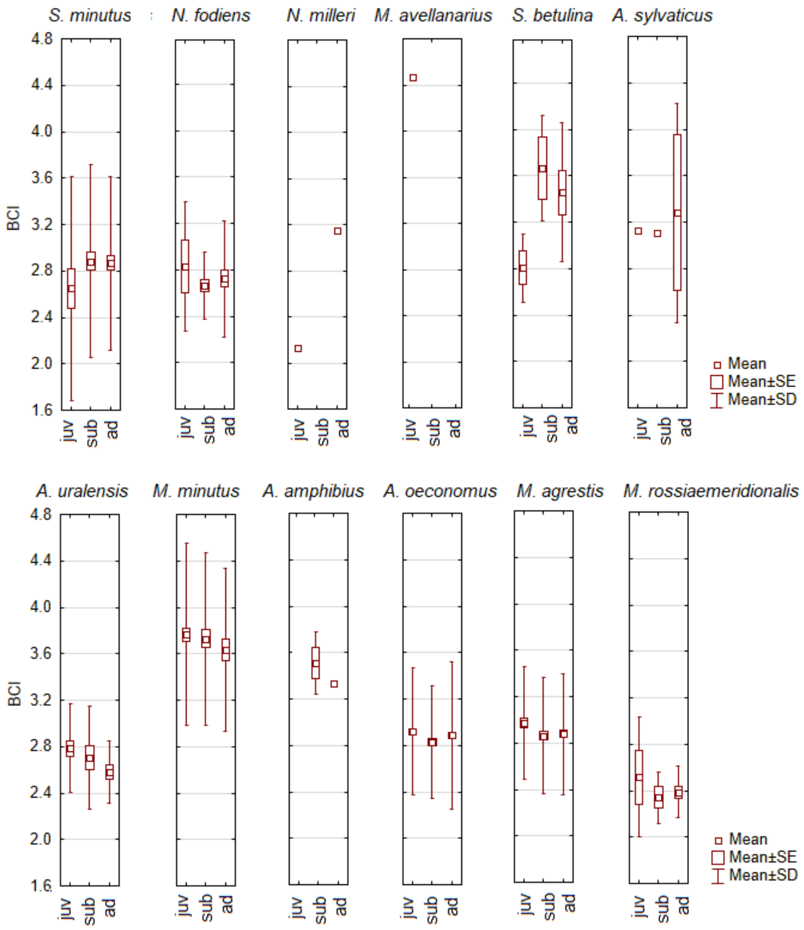

Figure S1. Non-significant age-related differences in body condition index (BCI) of small mammals.

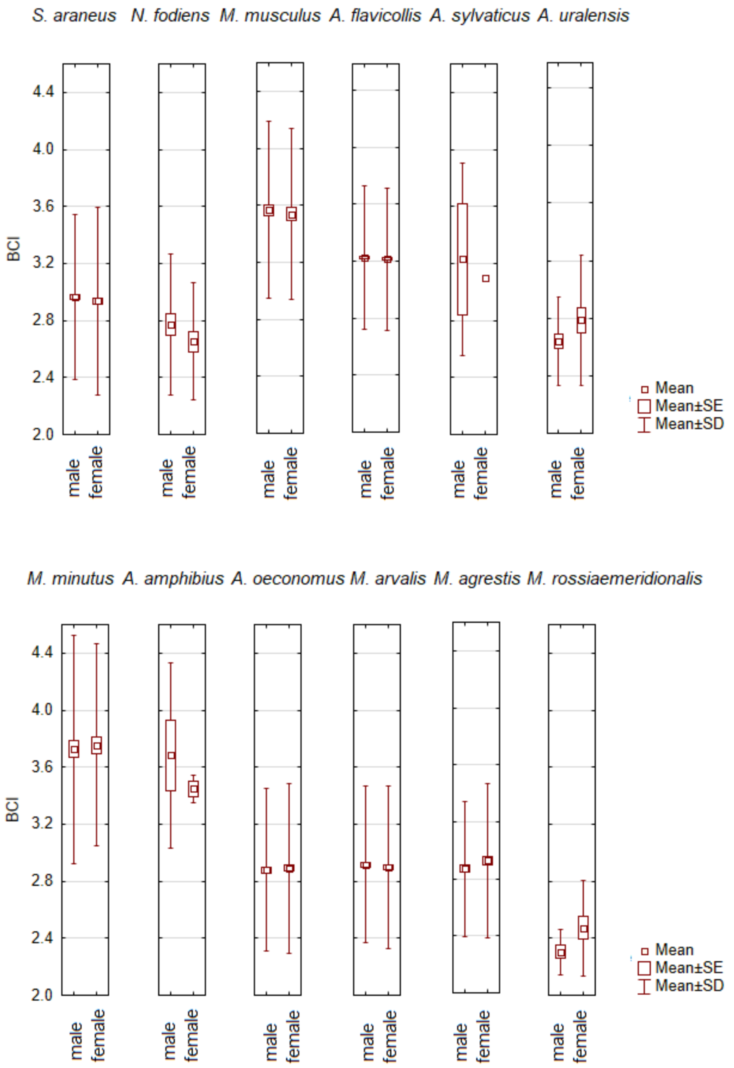

Figure S2. Non-significant gender-related differences in body condition index (BCI) of small mammals.

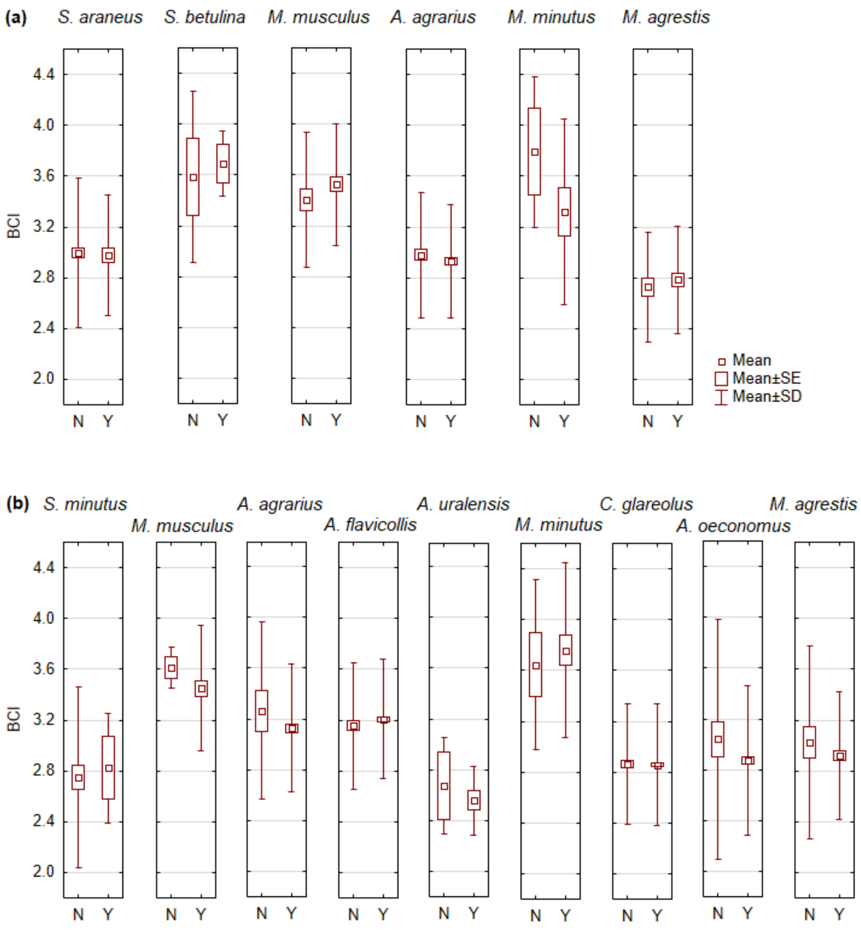

Figure S3. Non-significant reproduction-related differences in body condition index (BCI) between male **(a)** and female **(b)** small mammals: N – non-breeding, Y – breeding individuals. Note: cases with n < 5 not presented.

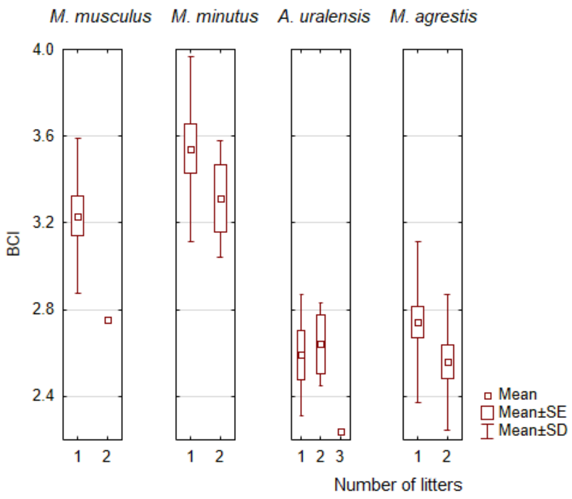

Figure S4. Non-significant differences in body condition index (BCI) related to the number of litters produced by females.

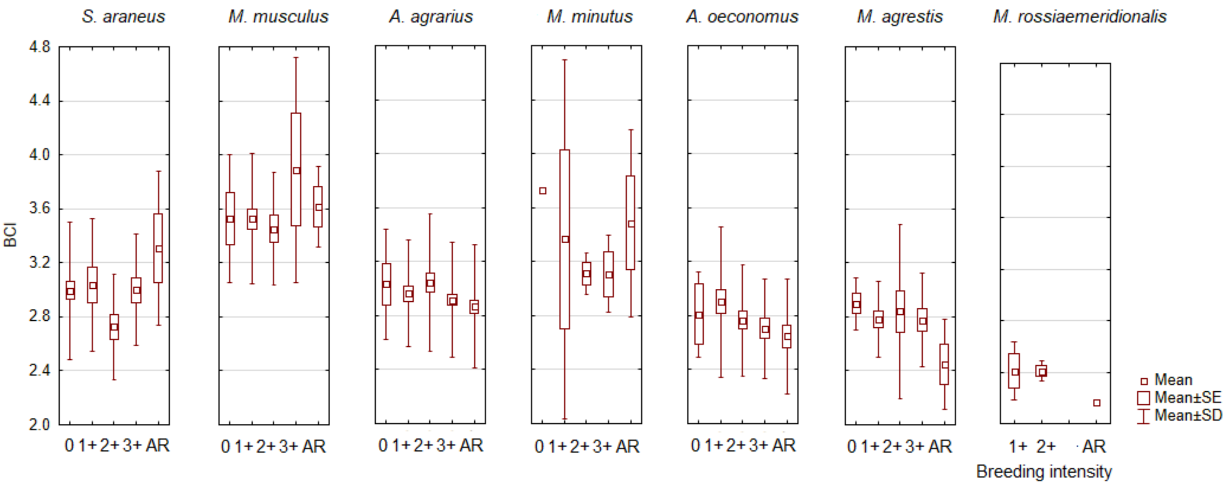

Figure S5. Non-significant differences in body condition index (BCI) associated with male breeding intensity: 0 – no spermatogenesis, 1+ – beginning of spermatogenesis, 2+ – spermatogenesis of medium intensity, 3+ – intensive spermatogenesis, AR – individuals after reproduction.
